# Fitness Landscapes of Functional RNAs

**DOI:** 10.3390/life5031497

**Published:** 2015-08-21

**Authors:** Ádám Kun, Eörs Szathmáry

**Affiliations:** 1 Parmenides Center for the Conceptual Foundations of Science, Kirchplatz 1, 82049 Munich/Pullach, Germany; E-Mail: szathmary.eors@gmail.com; 2 MTA-ELTE-MTMT Ecology Research Group, Pázmány Péter sétány 1/C, 1117 Budapest, Hungary; 3 Department of Plant Systematics, Ecology and Theoretical Biology, Institute of Biology, Eötvös University, Pázmány Péter sétány 1/C, 1117 Budapest, Hungary; 4 MTA-ELTE Theoretical Biology and Evolutionary Ecology Research Group, Pázmány Péter sétány 1/C, 1117 Budapest, Hungary

**Keywords:** origin of life, ribozyme, aptamer, RNA, fitness landscape

## Abstract

The notion of fitness landscapes, a map between genotype and fitness, was proposed more than 80 years ago. For most of this time data was only available for a few alleles, and thus we had only a restricted view of the whole fitness landscape. Recently, advances in genetics and molecular biology allow a more detailed view of them. Here we review experimental and theoretical studies of fitness landscapes of functional RNAs, especially aptamers and ribozymes. We find that RNA structures can be divided into critical structures, connecting structures, neutral structures and forbidden structures. Such characterisation, coupled with theoretical sequence-to-structure predictions, allows us to construct the whole fitness landscape. Fitness landscapes then can be used to study evolution, and in our case the development of the RNA world.

## 1. Introduction

A fitness landscape is traditionally defined as a function that assigns to every genotype a numerical value linearly proportional to its fitness. Since only phenotypes have fitness, therefore a fitness landscape is a mapping from genotype to phenotype to fitness. Genotypes can be defined on the level of alleles. Assume that there are only two alleles present for gene A: A1 and A2. A diploid individual thus could have three different genotypes: this is still a manageable genotype space. However, there are usually more than two alleles for any given gene, and there are many more genes even in the simplest bacteria. In the classical example of Sewall Wright [1] there are 10 allelomorphs in 1000 loci, which results in a space spanning 10^1000^ genotypes. Thus the main hurdle on the way to constructing fitness landscapes was already realized early in the development of evolutionary genetics: genotype spaces are vast. The size of the sequence space of an RNA or DNA of length *L* is 4^*L*^. In case of peptides, this space is then mapped (via the genetic code) to peptide space with size of 20^*L*/3^. Sequence space is in turn mapped to phenotypes. Some would argue that mapping genotypes directly to fitness (or some functional properties, like enzyme activity) is enough, as ultimately we want to arrive at a fitness landscape. However, the paths available to us are not that direct. If we could attach a fitness value to all sequences in genotype space, then our work would be done: we would have a fitness landscape. Usually, we can only sample some part of the sequence space and we need to infer the whole fitness landscape from this limited data. Extending this data to the whole landscape requires some assumptions, which will be discussed in this review. The genotype-phenotype-fitness mapping corresponds to sequence-structure-function maps in biopolymers. An often coveted goal is to understand the structure-to-function map, and thus how function changes with changes in the structure. 

Generally, a mutation can either change the structure or leave the structure intact. Changing the structure generally affects activity, except if the change happens far away from the active site or the substrate-binding parts. Mutations that do not affect the structure generally have little fitness effect [2,3], except when the exact chemical moiety present at the site is important for activity. We need to identify the **critical sites**, which are those where variation in the sequence destroys or severely decreases the activity of the molecule. *In vitro* generated mutants at these sites show low activity or no activity at all. Note that there are two kinds of critical site, and mutagenesis studies in themselves cannot distinguish between these two kinds. One kind we call a structural critical site, and the other kind a functional critical site. A **structural critical site** is important for the maintenance of structure; changes at this site would alter the structure extensively. A **functional critical site** contributes to function; for example, it binds the substrate, or lends a catalytic group to the active site. Change in a functional critical site might not alter the structure at all, but as the required chemical moiety is changed, it can no longer fulfill its function.

By identifying the critical sites and the structural features required for activity we can generalize the fitness landscape to areas of the genotype space not covered by mutants. In essence, a functional molecule has the right structural motifs (whatever their composition) and the right monomer at the functional critical sites. This generalization is usually missing, and unfortunately the data are not available to make this generalization. Without the generalization, we only have a list of genotypes and corresponding fitnesses for an insignificantly tiny part of the genotype space. This handful of genotype-fitness pairs might be located at and around a fitness peak (the wild-type sequence), but there could very well be many fitness peaks in the landscape and this particular one might not even be the highest [4]. If we accept that structure is an important determinant of phenotype and its knowledge is crucial for generalization, then we can state that RNAs lend themselves well to developing fitness landscapes.

**Figure 1:**
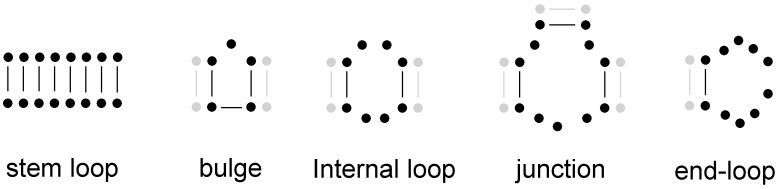
Secondary structure elements of RNAs. We can distinguish the following elements in a secondary structure: stem, bulge, internal loop, junction and end-loop. Nucleotides are depicted by a solid circle and hydrogen bonds by a line (except the horizontal line in the bulge, which is depicts the covalent bond between the nucleotides). The greyed-out nucleotides do not belong to the given structure. Note that the exact number of nucleotides in a bulge, internal loop, junction and end-loop can vary. Examples of secondary structures can be seen in Figure 2, Figure 3 and Figure 4.

The structure of an RNA can be approximated by theoretical calculations [5]. Such calculations are based on energetic parameters coming from experiments [6,7], and further refined by chemical probing and other biochemical analysis [6,8]. These approximation yields the so-called secondary structure of RNA (Figure 1). Secondary structure (or 2D structure) is the list of all intramolecular base-pairs the folded RNA harbours. The number of unique structures of this sequence space is estimated to be 2.35^*L*^ [9]. It is evident that there is by far fewer structures than sequences [9,10]. Current secondary structure predicting algorithms, for example the ViennaRNA Package [11], can predict more than 70% of the base pairs correctly based on an extensive set of known secondary structures [12]. The accuracy of these applications is constantly increasing [13], and they can predict the structure of longer sequences as well [14]. The function of the biological sequence ultimately depends on its 3-dimensional structure of which the secondary structure is only an approximation. Throughout the text we will mention instances when the secondary structure prediction needed to be amended because 3D interactions modified the structure. Such instances are examples where the usage of secondary structure as a proxy fails. The relative scarcity of these examples suggests that secondary structure is a good predictor of the 3D structure on which the function depends on.

The structure-function map can only be inferred by meticulous experimental analysis. Enzymatic activity or binding strength can be relatively easily measured, but knowing what certain parts of the structure do requires more investigation. In RNA viruses, like HIV, the underlying structure of the genome is important, as different sequences coding for the same protein (*i.e.*, synonymous mutation) can have different fitness [15,16]. Structural elements of the HIV genome, for example, were found to play role in slowing down translation so that the domains of the translated peptides can fold independently, and other structural elements allows frameshift during translation [17]. Understanding the fitness landscape underlying virus evolution [18,19] could help us predict their evolution and thus plan countermeasures in advance.

Fitness landscapes are not only theoretical constructs that might only be interesting to evolutionary biologist. They are also powerful conceptualization of the evolutionary potential of biological entities. Furthermore, they can also be used to design and engineer novel functional variants of biomolecules. Here we have focused on the fitness landscapes of RNAs. Aptamers and ribozymes are now recognized as having therapeutic potential [20,21,22,23,24,25] in a wide array of diseases from virus infections [26,27] to cardio-vascular diseases [28]. They can also be used as biosensors [29] and in drug delivery [30,31,32]. Understanding of RNA structure–function landscapes not only gives us more insight into the development of the RNA world [33] but is of applied significance, too.

Fitness landscapes of microbes [34], peptides [35], viruses [36], and RNAs [37] are available to some extent. A recent review of fitness landscapes concluded that while we have begun to appreciate the need for such landscapes, especially as they turn out to be rugged and not smooth, we are only beginning to collect the required data [38]. Here we would like to review as well as extend what we know about the fitness landscapes of aptamers and ribozymes (functional RNA). Furthermore, we would like to highlight areas badly in need of more data.

## 2. Whole Genotype–Fitness Map

A full fitness landscape requires a map from every possible genotype to fitness. The realization of every possible genotype is usually technically not feasible due to the combinatorial explosion of the genotype space. Library sizes employed in SELEX [39,40,41,42] are about 10^13^–10^15^, which can cover the whole sequence space of up to 25mers. Thus, for very short sequences, the genotype space can be realized and by subsequent selection probed. We need to appreciate this possibility! A fresh 1 TB HDD can only hold a tiny fraction of this information (2.5 × 10^11^ positions if we store each nucleotide in 2 bits, so each byte stores 4 nucleotides). This is a perfect example of when “wet” computers can store more information per volume or mass, and do more computations per unit time (*i.e.*, evaluation of function) than digital computers.

The possibility of scanning a whole genotype space was realized in Irene Chen’s lab [43]. They have started from a pool of nearly all 24mers (there are 281,474,976,710,656 different sequences) in thousand-fold excess. Then they selected for GTP binding. The aptamers were sequenced and thus the resulting fitness landscape maps sequences to GTP binding strength. As expected, there are peaks in the landscape around which the functional sequences congregate [43]. Based on the results we can infer some general characteristics:

(1) *There are multiple solutions to being a GTP aptamer.* The fact that there is neither a common sequence motif nor a common structural motif that would tie together the fitness peaks suggests that there are multiple, independent solutions to a GTP aptamer. Thus the sequence space can be populated with different aptamers that have nothing in common except that they have the very same function.
(2) *There are uncommon structural solutions as well.* In order to assess how frequent or infrequent the structures reported in S14 of the paper by Jiménez *et al.* [43] were, we folded 1 million random sequences of length 24 and estimated the frequency of the common structures from this sample. We know that this is a tiny fraction of the 2.814 × 10^14^ unique 24mers, but the common structures should be well represented even in this small sample. The structural repertoire of 24mers is rather limited. The structural space is dominated by the unstructured structure with roughly 18% of the sequences folding into this structure. Four of the peaks were found to be unstructured according to the Vienna Package. We cannot exclude the possibility of such a structure to bind the ligand, but it is more likely that an induced fit mechanism determines the final structure, or that the structure is stabilized by interactions that cannot be predicted by the employed 2D folding algorithm. Most of the other peaks were also folding into quite common structures (Table 1). A common structure is defined as those structures that are formed by more sequences than the average structure [44], *i.e.*, *N_c_* > 4^*L*^ / *S_L_*, where *N_c_* is the number of sequences folding into a common structure, *S_L_* is the number of distinct structures of length *L*. Most of the sequences fold into one of the common structures [44]. For example, more than 90% of the 25 nt long sequences comprising only G and C nucleotides fold into a common structure. Consequently, while there could be many rare structures, very few sequences fold into them. However, there was one peak (m20j22) with a structure that we have not found in our sample of structures for 10 million sequences. We can safely assume that this structure is uncommon. While we expect that evolution will mostly (if not always) find the common structures, it is reassuring to know that uncommon structures can also have function, just they are unlikely to be found by evolution. Aptamers and ribozymes we know are all folding into common structures not because only common structures can have function, but because they are the ones that can be reached by evolution.

**Table 1:** Structures and their frequencies among common structures of fitness peaks of a GTP aptamer.

Structure ^1^	Peaks folding into this structure ^2^	Frequency in our sample ^3^	Frequency in our larger sample ^4^
........................	m18j13, m14j12, m04j04, m02j01	18.7%	18.8%
((((....))))	m19j09	3.4%	3.4%
(((......)))	m15j18, m10j11, m07j07	2.4%	2.5%
((((...))))	m16j20	1.8%	1.9%
((((.........)))	m17j08	0.8%	0.8%
(((..........)))	m01j03	0.6%	0.6%
((((.......))))	m03j02	0.4%	0.4%
((.((....)).))	m05j10	0.3%	0.3%
(((((.(.....).)))))	m06j06	0.05%	0.006%
((.....))...(....)	m20j22	not found in our sample	not found in our sample

^1^ We assume that leading and trailing unpaired bases are not important, and they were left out; ^2^ Naming of the peaks is as in the original paper; ^3^ Based on 1 million random sequences; ^4^ Based on 10 million random sequences.

## 3. Lessons from Aptamers

The landscape described previously is an important milestone in the study of fitness landscapes. Strictly speaking it represents a genotype-to-fitness map, without too much reference to the phenotype. Knowing the structural and sequence (phenotype) requirement of a GTP aptamer would allow us to answer questions like “Is there a sequence that need to be in the loop in order to bind GTP?”, “Is there a minimum stem-loop length for the aptamer?” or “Is there any structural significance to the internal loop in m05j10 or m06j06?” Selection of longer aptamers followed by deep-sequencing and structure probing could provide us with insight, albeit at the expense of looking at the whole sequence space. Aptamer research, due to relatively shorter sequences and easier selection, has offered some further insight into the structural requirement of certain functions. Most studies on aptamers or ribozymes only report the selected sequences [45,46,47] though some report the folded structures [48,49] as well. Studies that actively try to deduce a consensus structure [50,51] lead us one step further in understanding structure–function relationships. Recent studies go even further and implement the consensus in order to obtain the minimum motif(s) required for activity.

**Figure 2:**
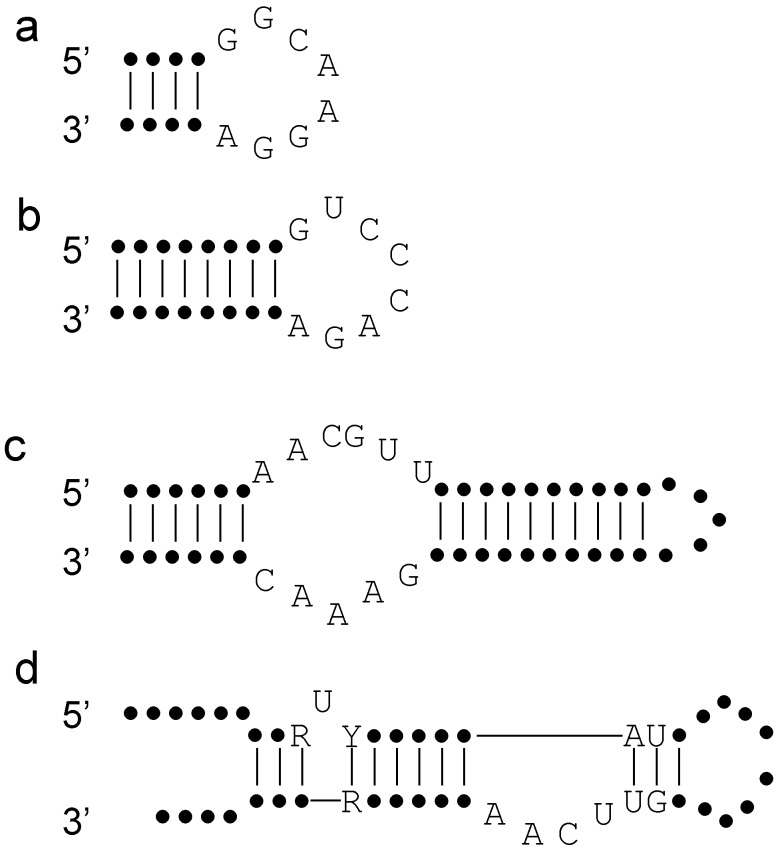
Consensus structures of aptamers selected against HIV-1. Dots represent nucleotides without sequence specificity. Vertical lines represent hydrogen bonds, whereas horizontal lines represent the link between nucleotides (these links are omitted between nucleotides close to each other of the figure). (**a**) Aptamter against the 5′-UTH region of HIV, (**b**–**d**) aptamers against the reverse transcriptase of HIV.

For example, aptamers selected to bind to the 5′-untranslated region of the HIV have a common structural element and a common sequence motif: the “*consensus octamer* GGCAAGGA *is always placed in an apical loop flanked by complementary sequences that form a double-stranded region of at least 4 bp in length*” [52] (Figure 2a). In the *in vitro* selected aptamers (64 nt) this motif could be found in various contexts (see Figure 4 in [52]), but an engineered minimal loop with the right structure and sequence can also inhibit HIV. A binding aptamer against the trans-activation responsive (TAR) RNA part of HIV-1 also demonstrate that sequence complementarity in an end-loop is not sufficient to ensure functionality [53]. The requirement for a binding aptamer was found to be “*(1) a loop complementary to the entire loop of the target RNA, (2) a double-stranded stem region comprising generally more than 4 bp adjacent to the loop, and (3) a closing GA pair*” [53] (Figure 2b). These aptamers contain perfect examples of critical sites: certain sequences need to be located in the right structural context. Changing either the sequence or the structural context can ruin the aptamer.

A similar result was obtained for the aptamer that binds to the reverse transcriptase of HIV: the strongly binding (6/5)AL aptamer needs to have an internal loop of 6 nucleotides on one side, and 5 on the other, flanked by at least 6 base pairs on one side and 10 on the other (Figure 2c) [54]. The flanking stems can be longer, and there could be slight variation in the sequence of the internal loop. Which stem ends in a loop, and which bears the start/end of the sequence, does not seem to matter. Another aptamer selected against the same target show a consensus structure which is a “*simplified stem-loop structure in which the UCAA bulge is flanked on one side by two highly conserved base pairs (AC/GU) and on the other by a largely generic helix that is interrupted by a single unpaired U residue*” [55] (Figure 2d). While in-depth analysis of the structure showed that there might be other requirements for an active inhibitor (functional aptamer), the UCAA bulge and the flanking base pairs are a constant feature, and quite a number of other structural elements can be changed without loss of function [55].

The consensus structures/sequences of Figure 2 are sufficient to ensure aptamer binding, but they are certainly not the only structures that are functional. The studies [52,54,55] demonstrate that a small motif embedded in a larger structure can be functional. Thus (especially for aptamers) we need to look for small motifs that are common to the selected structures, and there is no need to strictly adhere (pun intended) to maintaining much larger structures. 

## 4. Lessons from Ribozymes

Aptamers usually bind via a stretch of well-defined nucleotides, and because of their relatively short length they cannot have too rich a structure. Quite a few aptamers are made of an end-loop with a small stem. This is only as far as we can go with the study of aptamers in our quest for a fitness landscape. Ultimately we would like to understand the fitness landscapes of enzymes (genes). As ribozymes are generally around 50–300 nucleotides long (very small ribozymes are also known [56,57]), their whole genotype space cannot be produced. We can only hope to study the mutational neighbourhood of the wild-type sequence. A sequence of length *L* has *3L* single point mutants, *i.e.*, mutants that only differ at one position. For large sequences, this number of mutations can also be quite high, not to mention that the fitness of the sequences have to be characterized as well. This is not unfeasible either, as demonstrated recently for the *E. coli* TEM-1 *β*-lacatmase. Nearly all single-point mutants in genome space (2536/2583) and in protein space (15,167/18,081) were generated and their fitness was estimated [58].

Still, there are very few ribozymes studied in enough detail for any meaningful inference of a functionality landscape. For the most part, we can only consider the natural ribozymes [59]. Unfortunately even among these few ribozymes some need to be excluded for practical reasons: the Hepatitis Delta Virus and like ribozymes [60] and the RNase P [61] have a pseudo-knot [62], a structural element that cannot be computed by the most widely used secondary structure-predicting algorithms. This is also true for the well-studied artificial Class I ligase [63] and derived ribozymes (e.g., the RNA dependent RNA polymerase ribozyme [64,65,66,67]). Enough data might not be available for the more recently discovered natural ribozymes like the *glmS* ribozyme [68], and the twister ribozymes [69]. This leaves the Group I [70] and II introns [71], *Neurospora* VS [72], the hairpin [73], the hammerhead ribozyme [74], the Diels-Alderase ribozyme [75] and the class II ligase [76] for consideration.

The Class II ligase would be the perfect subject for a fitness landscape: it is short (54 nt) and does not have a pseudo-knot. Furthermore, and more importantly, each of its nucleotides was replaced by the other 3 nucleotides not present at the given site, and activity was measured [77]. There is a catch, however: even the fittest mutant has an order of magnitude less activity than the master sequence. That is very unusual, as some part of the ribozyme is probably not that important, and some sequence variations are generally tolerated. The activity of the wild type is described in another paper [78]. The methodology employed to assay enzymatic activity was different in the two studies, and thus it is not surprising to find different activities for the same mutations (Table 2). This raises the question of whether the activity of the wild-type ribozyme would be the same in the second study. The activities highly correlate (linear fit with R^2^ = 0.976), but the slope is mainly determined by the activities of mutations G2U, G2A, G2C and G49 (and the data points are not independent). According to this admittedly flawed statistic, the activity of the wild-type sequence should be 3.7 times lower in the second study. However, the wild-type ribozyme still exhibit orders of magnitude higher activity than any of the mutants. Such a high fitness peak compared to its mutational neighborhood (*i.e.*, mutants differing at one site) is interesting, and merits further study.

**Table 2:** **Activities of mutants of the Class II Ligase.**

Mutation of the Class II Ligase	Activity from [77]	Activity from [78]
G1A	0.00173	3.80 × 10^−4^
G2A	0.03043	1.10 × 10^−1^
G2C	0.00599	3.50 × 10^−2^
G2U	0.03584	1.30 × 10^−1^
A3G	0.00147	6.30 × 10^−4^
A3C	0.00108	8.00 × 10^−5^
A3U	0.00134	1.60 × 10^−4^
G49A	0.01048	2.10 × 10^−2^
G47A	0.00111	2.00 × 10^−5^
G47C	0.00089	1.90 × 10^−5^
G47U	0.00108	9.50 × 10^−6^

**Figure 3:**
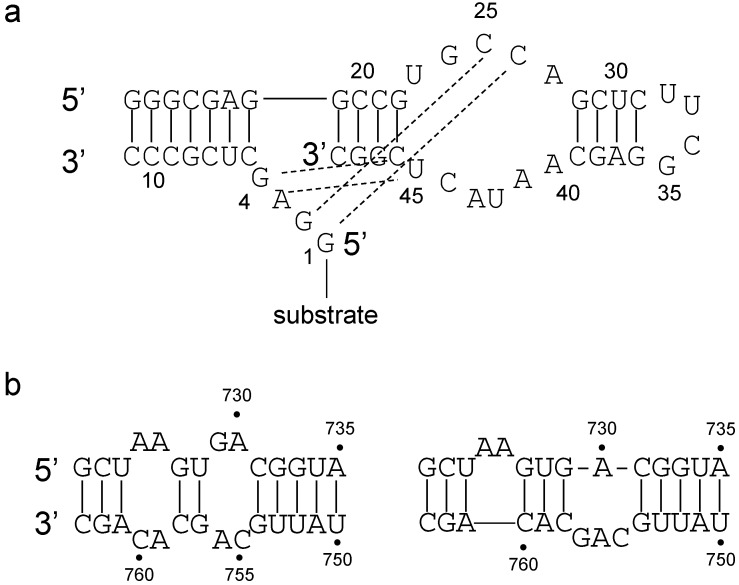
(**a**) The Diels-Alderase ribozyme’s secondary structure. Numbering is as in [82]. Dashed lines represent hydrogen bonds that are present but not predicted by conventional secondary structure predicting algorithms. (**b**) The predicted secondary structure of the VS ribozyme according to folding algorithms and NMR (**left**) and the active conformation determined by chemical probing (**right**).

The core part of the class II ribozyme (the 3-way junction and arms P2 and P3) was predicted well by the Vienna RNA package [11,79]. Unfortunately, the P1 stem containing the template to which the substrate is hybridized cannot be predicted well. Such a discrepancy between the predicted and the chemically determined secondary structure hints at non-canonical intramolecular interactions. For example, the 49mer Diels-Alderase ribozyme’s [80] predicted secondary structure has a base pairing between G26-C44 (Figure 3a, this pairing is not shown). However, the GGAG sequence, which actually has the substrate tethered to it, binds with bases in the internal loop (Figure 3a, hydrogen bonds with dashed lines) [81]. Thus the G26-C44 pair cannot form. Similarly for the VS ribozyme, the secondary-structure predicted by RNAfold [11] and the one predicted by chemical probing [82] are different. NMR studies [83] corroborated the theoretical secondary-structure with the addition that the active site harbours a number of non-canonical base-pairs (A^+^-C, G-A and U-G). This structure is probably the ground-state structure [83], which then transforms into the active structure determined by chemical probing [82] (Figure 3b).

Thus, while the secondary structure can be a good proxy for the 3-dimensional structure, some interactions out of the plane or the interaction with the substrate can modify the structure.

**Figure 4:**
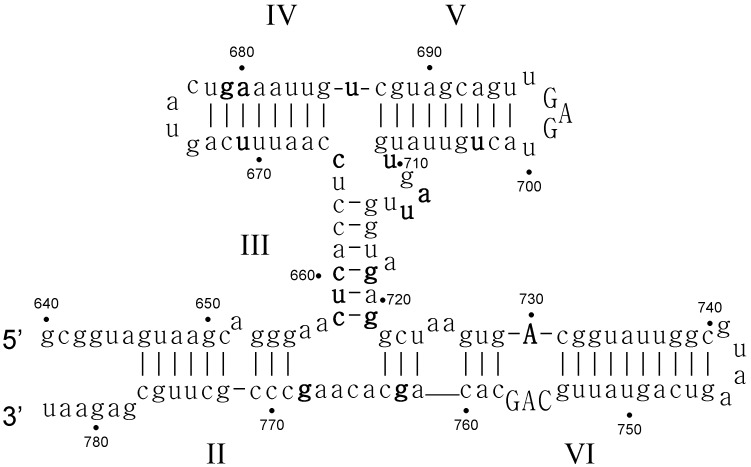
Secondary structure of the *Neurospora* VS ribozyme. Numbering is according to convention [82]. Positions in bold represent structural critical sites. Capitalized positions represent functional critical sites. (A730 is both).

We have constructed a functionality landscape (used as a proxy for fitness) for the *Neurospora* VS ribozyme and the hairpin ribozyme [84,85]. Here we discuss the fitness landscape of the VS ribozyme in more detail. The VS ribozyme is a moderately sized ribozyme with a rich structure (Figure 4). The full 3D structure of the ribozyme is not yet available, but NMR has been extensively employed to study important localities of the ribozyme [83,86,87,88,89,90,91,92,93,94].

Basically, the end-loop of region V binds the substrate, so that the internal loop in region VI can align with the cleavage site. Structurally this means that the length of region V [95,96,97], the linker region III [95] and the part of region VI between the J236 junction and the active site internal-loop are important. In the case of region V, it needs to be the right length or at most one base-pair shorter or longer [97]. As it needs to align with region I (the substrate), a change in the substrate (increase in the length of region Ib) necessitates a decrease in the length of region V [97]. The length of the rest of the stem-loops can be varied in the *trans*-acting ribozyme [95,98]. We note here that the length of region II was not varied; this part is generally considered to be unimportant for the *trans*-acting ribozyme. It would still be nice to know for sure. However, the bulge at position 652 in stem-loop II is required, though its chemical nature is not important [82,98]. Activity decrease to about fifth of the wild-type value for A652C and A652G, but the activity of A652G is one tenth that of the wild-type ribozyme. The former mutations do not change the predicted secondary structure, while the latter does (albeit causing only local rearrangement). We know that pairing of this bulge does not affect activity much [82,95], but removal of the bulge or putting it on the other side of the stem-loop does. Similarly, changing the nucleotides of the bulge at positions 725/725 in region VI does not change activity (actually, it increases activity, except for the A725G mutation which also changes the structure), but removal of the bulge decreases activity [98]. The above is generally true for the bulge at position 718 in region III as well. No data is available for change of nucleotide, but removal and inversion of the bulge has the same deleterious effect [95] as previously. The predicted secondary-structure would only change for A718U, and then only slightly.

The junction 3-4-5 contains a number of critical sites. The nucleotides U710/G711/A712 form a canonical U turn (5′-UNR-3′ R = A or G) [99]. Thus at position 711 there could be any ribozyme, and indeed changes at these site are neutral. The A712C and A712U mutations would interfere with the U turn, and they have low activity (though they do not change the predicted secondary-structure). Interestingly, the A712G mutation has a low activity, though the U turn could theoretically form. But this mutation also rearranges the minimum free energy structure, and thus it has a low activity. Changing the position 686 again changes the predicted secondary-structure, and accordingly the U686A mutation has low activity (activity for U686C and U686G is not available). Similarly, C665G and C665A decrease activity and change the structure. Unfortunately the activity of C665U is not available, as it might not decrease activity, since the predicted secondary-structure does not change. Thus the positions 710, 712, 686 and 665 are structural critical sites.

The catalytic site of the ribozyme is the internal loop in region VI of the ribozyme [98], especially the adenine at position 730. The nucleotides C755, A756 and G757 are also important, and most of their mutations change the structure as well. The A730 nucleotide is both a critical functional site and a critical structural site.

According to the above we can distinguish four kinds of structural elements:

- **Critical structure** contains functional critical sites. Figure 2 shows a number of such structural elements, and the end loop of region V and the internal loop of region VI of the VS ribozyme are further examples.
- **Connecting structure** contains no functional critical sites, but it has structural critical sites, and is important for the positioning of the functional critical sites. Region III is such a structure: it connects the substrate binding region V and the active site region VI.
- **Neutral structure** is the part of the ribozyme that can be freely changed, even removed. Region IV and II, and the distal part of region VI are examples of such structures.
- **Forbidden structure** is one that would decrease or abolish activity if present. Examples are the inverted bulges in region II, III and IV of the VS ribozyme. The information content of absence is often overlooked [100]. For a general fitness landscape we also need to know what can be there and what cannot.

The substrate region (region I) of the VS ribozyme has received some focus in recent years. For example, the G638 site in the substrate loop is also a functional critical site. The structure of the G638A mutant is the same as the WT substrate, yet the catalytic rate is nearly 4 orders of magnitudes lower. “The results suggest that the main requirement for the nucleobase at position 638 for cleavage under standard conditions is the C6 carbonyl, or the imino proton on N1, or both” [101].

We have folded all possible point mutants of the VS ribozyme (Supplementary Table S1) in order to determine structural critical sites. Sites at which the predicted secondary structure changed considerably if a point-mutation was introduced are potential structural critical sites. The mutations A648C, C773A, U703A, U703G, U705A, G694A, A680C, and G679C change the predicted structure at more than 34 places, which translates to considerable structural rearrangements. Unfortunately no activity data is available for any of these sites.

## 5. Double Mutations and the Question of Epistasis

The local view of a fitness landscape usually falls short when one needs to assess the effect of multiple mutations. We mostly know what happens to the ribozyme (or gene, or organism) if we introduce one mutation; unfortunately there is hardly any data on the effect of multiple mutations. People might say that there is a huge amount of literature on epistatis [102]. This is true, but most studies on epistasis (or any interaction of mutations) only record the genotype and the resultant fitness effect.

Suppose we introduce two mutations into a sequence. What will happen? Based on the mutational neighbourhood of ribozymes and other sequences, we can safely say that if one of the mutated sites is a critical site then activity is abolished. Can we say anything more? Can we say that if none of the mutation affects activity then activity will not be affected? No, we cannot. The minimal free energy structure can very well change, and the whole sequence can fold into a very different structure, even if the individual changes do not affect activity. Can we say that if both mutations only slightly affect fitness, then they both together will again only slightly affect fitness (maybe to a slightly greater extent than the individual ones)? Again, we cannot. For example, restoring a base pair usually restores activity. Mutations of base pairs are thus examples of sign epistasis [103], in which the individual mutations decrease activity but both together restore (increase) activity. Sign epitasis is usually defined the other way around, *i.e.*, substitutions that are beneficial individually, but lethal or very deleterious together [104,105,106,107,108].

If we want to make some generalizations about the effect of double and multiple mutations, we should treat base pairs differently. We suggest that base pairs should be considered *one* unit of an element in a structure, and not as two independent bases. A given base-pair can either mutate into a mispair (usually lowering or abolishing activity) or mutate into a base-pair, in which case activity does not necessarily change. There are 47 base-pairs in the *cis*-acting VS ribozyme (Supplementary Table S1). Out of these base pairs, only 6 were such that changing the base pair to a canonical base pair changed the predicted secondary structure. In a further 11 cases, the structure changes if the base-pair is changed to a GU or an UG base-pair. Otherwise (30 cases), the secondary structure does not change if the base-pair is changed to any of the other possible base-pairs. However, not all such predicted neutral changes are actually neutral (Supplementary Table S1). A mispair (an open base-pair compared to wild-type structure) usually has a mild deleterious effect on activity [85]. More than two mispairs in the same region surely abolish activity, though, which again demonstrates that the structure can slightly vary, but too much structural change leads to an inactive ribozyme. The base-pairs flanking the junctions are, however, critical. In some cases, changing them to other base-pairs also changes the structure (658:721 CG→AU; 663:715 CG→GU; 655:769 GC→UG). Thus structural critical sites can be generalized to **structural critical units**, encompassing critical base-pairs.

Outside the realms of base-pairs and critical sites, there could still be double and multiple mutations whose effect we need to know in order to characterize a fitness landscape. We do not expect complex epistatic interaction between the sites; if there were some, then at least one of the sites would be a critical site. The most common assumption is a multiplicative (additive) fitness effect, in which case, if mutants individually have, for example, half the activity of the wild type, then the double mutant would have 25% activity (0.5·0.5). An early and pioneering study by Lehman and Joyce [109] corroborated this expectation for the *Tetrahymena* ribozyme. Since then others also found either multiplicative fitness effects or some synergistic effects, in which cases the double mutant has higher activity than expected by the multiplicative model. Such studies include the study of the RNA virus *Tobacco etch potyvirus* [110]; the study of kinase ribozymes [111,112]; and our own collection of mutants of small, natural ribozymes [85]. In conclusion, the easiest way to deal with multiple mutations is to assume mutational independence (multiplicative effects), although it slightly overestimates the decrease in fitness due to multiple mutations.

We still hasten to add that more data on triple and higher order mutants as well as systematic investigation of multiple mutations of known ribozymes would greatly help us to assess the combined effects of multiple mutations.

## 6. Far from the Wild-Type Region of the Landscape

We know more and more about the immediate mutational neighbourhood of RNA viruses, aptamers and ribozymes. This alone allows us to build better representations of fitness landscapes. The ultimate goal is to also know the fitness landscape far from the wild-type sequence. To our knowledge no such investigation has been conducted. We know that there are multiple fitness peaks [43] and that the same secondary structure can be realized with very different sequences [10,113]. Hayashi *et al.* [114] have randomized a part of the fd phage, thereby abolishing infectivity. They then introduced random mutations and measured infectivity. Fitness increased smoothly to about 40% the original level, but then the ruggedness of the landscape became dominant and evolution slowed down. The peptide evolved to a sequence different from the wild-type, thus arriving at another fitness peak. This means that there are fitness peaks of equal height away from the original one. We do not know if these sequences were structurally similar to the original structure (and this was on a peptide anyway). The studies discussed in the section on aptamers (see above) are informative here as well: they demonstrate that as long as core structural/functional elements are present, other parts of the aptamer can change without loss of activity. We can therefore assume that the fitness landscape has a similar structure surrounding functional but sequence-wise different (as much as sequence can differ, c.f. functional critical sites) sequences.

## 7. Summary

- There are multiple solutions to the same functionality.
- Functionality often depends on small motifs, and the rest of the aptamer need only not interfere with the motif or to allow the required stacking of motif parts.
- Even very rare structures can have a function, though evolution will rarely, if ever, find them.
- There are critical sites where substitutions are not tolerated. These sites can be either functional critical or structural critical sites/units. Change in a functional critical site does not change the structure of the RNA, but abolishes activity. Change in a structural unit (a single nucleotide or a base-pair) abolishes activity through change in the structure.
- Structural elements or bases not appearing at certain places are as informative as permitted structures. While these structural elements, by definition, are not part of the structure, they show the limit of structural changes. We call these features forbidden structures.
- Apart from critical and forbidden structures, a structure can be characterized by uncovering connecting structures and neutral structures too. Connecting structures link the structural critical sites, but their exact forms are not prescribed. Neutral structures can be changed (to some extent) freely without affecting activity.
- RNA structure is even important for peptides, as the structure of the mRNA or RNA viruses can influence the replication, expression and activity of the translated peptide even when the resultant peptides would have the very same sequence (*i.e.*, there are only synonymous changes).

## Supplementary Files

Supplementary File 1
